# Prognostic value of 48-h reassessment of a laboratory-derived frailty index in ICU patients with sepsis: model development in MIMIC-IV and external transport evaluation

**DOI:** 10.3389/fmed.2026.1926236

**Published:** 2026-09-23

**Authors:** Ming He, Yuxuan Zhang, Jiujia Xiao, Mengjiao Yang, Yi Wen, Dan Huang

**Affiliations:** Department of Critical Care Medicine, The First Affiliated Hospital of Chengdu Medical College, Chengdu, Sichuan, China

**Keywords:** 48-h reassessment, calibration, electronic health records, external transport, FI-LAB, laboratory-derived frailty index, MIMIC-IV, sepsis

## Abstract

**Background:**

Static laboratory-derived frailty index (FI-lab) measures have been associated with adverse outcomes in sepsis, but the value of reassessing acute multisystem laboratory abnormality burden after initial resuscitation and transporting a prespecified model to a new clinical setting remains uncertain.

**Methods:**

We conducted a retrospective 48-h landmark study using MIMIC-IV for model development and an independent single-center ICU sepsis cohort from January 2020 to December 2025 for supportive single-center transport evaluation. FI-lab-23 was calculated from 23 routine laboratory variables using prespecified thresholds, with at least 17 components required, and was interpreted as an acute laboratory deficit-accumulation index rather than chronic frailty. The primary extended model added FI-lab-23 measured at 24–48 h to clinical variables; the strict secondary model additionally included lactate clearance. Fixed-horizon logistic regression predicted 28-day mortality among patients alive and remaining in the ICU at 48 h. MIMIC-IV coefficients were frozen and applied without refitting to the local cohort. Performance was assessed by discrimination, Brier score, calibration, and exploratory decision-curve analysis. Baseline/change parameterizations were treated as secondary specification analyses; sensitivity analyses addressed testing intensity, component completeness, and a fixed 10-component common-core FI.

**Results:**

The primary MIMIC-IV development cohort included 6,153 patients with 1,594 deaths. Each 0.05 increase in 24–48 h FI-lab-23 was associated with higher 28-day mortality (OR: 1.178, 95% CI: 1.149–1.207). Optimism-corrected AUC was 0.710. Of 722 local landmark-eligible patients, 328 met the primary complete-case requirements (85 deaths). In this selected cohort, the frozen model achieved an AUC of 0.774, Brier score of 0.160, calibration intercept of 0.109, calibration slope of 1.273, and observed-to-expected ratio of 1.074. Baseline FI-lab did not improve fit after the 48-h level was known, and baseline-by-change interactions were not significant. The direction of association was preserved across sensitivity analyses, although effect magnitude varied with component-completeness criteria.

**Conclusions:**

FI-lab-23 reassessed at 24–48 h provided updated prognostic information for 28-day mortality in ICU patients with sepsis. The frozen MIMIC-IV model showed encouraging but imprecise performance in a selected single-center complete-case cohort. These findings constitute supportive transport evidence rather than definitive external validation, and larger multicenter studies are required before clinical implementation.

## Background

Sepsis is life-threatening organ dysfunction caused by a dysregulated host response to infection and remains a major global cause of death (1, 2). Contemporary management emphasizes early recognition, timely treatment, and repeated reassessment because risk evolves rapidly during the first days of critical illness (3). The Sequential Organ Failure Assessment (SOFA) score quantifies established organ dysfunction (4), whereas lactate and lactate clearance provide information about hypoperfusion and response to resuscitation (5–7). Neither approach fully represents the multisystem laboratory abnormalities produced by immune dysregulation, endothelial injury, coagulation disturbance, hepatic and renal stress, electrolyte imbalance, and altered metabolic reserve (8, 9).

The frailty index is based on accumulation of health deficits (10, 11). Laboratory-derived frailty indices extend this principle to routinely collected tests and have been associated with mortality across adult populations (12, 13). In the present acute sepsis setting, FI-lab-23 is used as a laboratory deficit-accumulation index and should not be interpreted as a validated measure of chronic geriatric or baseline biological frailty. Recent studies have linked admission FI-lab measurements to adverse outcomes in septic shock and older patients with sepsis (14, 15). However, these investigations were predominantly static. Smaller recent studies have also suggested that reassessment of individual biomarkers at approximately 48 h may provide information beyond admission SOFA (16, 17), but they did not evaluate a prespecified multicomponent FI-lab model with locked external transport.

A change score is not automatically a distinct biological construct. Because ΔFI-lab equals FI-lab at 48 h minus baseline FI-lab, a model containing baseline FI-lab and ΔFI-lab is algebraically equivalent to a model containing baseline and 48-h levels. Claims that an identical change has different implications at different baselines require an explicit baseline-by-change interaction rather than main effects alone. Change scores are also vulnerable to mathematical coupling, regression to the mean, and changes in the set of tests measured (30). Accordingly, the present revision treated FI-lab-23 at 24–48 h as the primary updated predictor and evaluated baseline-plus-change and interaction models only as secondary specification checks.

We first aimed to determine whether a reproducibly defined FI-lab-23 measured at 24–48 h was independently associated with 28-day mortality after initial resuscitation. Separately, we evaluated whether adding the 48-h FI-lab-23 measure improved model fit and discrimination beyond the prespecified clinical model and characterized calibration and exploratory decision utility after frozen-coefficient transport to an independent local ICU cohort. Baseline-plus-change, baseline-plus-T1, and interaction models were treated as secondary specification analyses; testing-intensity and common-core analyses assessed robustness.

## Methods

### Study design, data sources, and reporting

This retrospective prognostic model study used a development-and-transport design. The development cohort was derived from MIMIC-IV, a deidentified electronic health record database from Beth Israel Deaconess Medical Center (18, 19). The local supportive transport cohort included adults admitted with sepsis to the ICU of The First Affiliated Hospital of Chengdu Medical College between January 2020 and December 2025. The study was reported according to STROBE and the updated TRIPOD framework, with external evaluation principles guided by contemporary recommendations for clinical prediction models (20–24).

### Study population and 48-h landmark

Adults aged 18 years or older with an established diagnosis of sepsis according to Sepsis-3 were eligible. In both MIMIC-IV and the local database, Sepsis-3 criteria were applied retrospectively during cohort construction. Only the first eligible ICU admission per patient was retained. ICU admission was the study time origin, and all baseline and reassessment windows were defined relative to that time point. The prediction time was the 48-h landmark. Patients had to be alive and remain in the ICU at the 48-h landmark. The outcome was the recorded all-cause 28-day mortality status from ICU admission, conditional on survival and remaining in the ICU to the landmark. In the local cohort, 28-day mortality was determined from the prespecified mortality-status field; deaths recorded within 28 days after ICU admission were counted as events even when they occurred after transfer from the ICU to another hospital ward. Completeness of post-discharge vital-status ascertainment could not be independently verified.

The MIMIC-IV source cohort contained 20,962 eligible patients. For the common specification-analysis population, 16,924 had at least 17 FI-lab-23 components at T0 and 7,952 had sufficient components at both T0 and T1. Among these, 241 died by 48 h and 1,229 were discharged from the ICU before 48 h, leaving 6,482 patients at the landmark. The local source cohort contained 952 patients; 11 died by 48 h and 219 were discharged from the ICU before 48 h, leaving 722 landmark-eligible patients. Among these 722 patients, 624 had at least 17 T0 components and 343 had at least 17 components at both T0 and T1. Fifteen of the 343 lacked admission lactate, leaving 328 patients with 85 deaths for the primary transport analysis; a further 105 lacked repeat lactate, leaving 223 patients with 66 deaths for the strict lactate-clearance analysis. The fixed 10-component common-core analysis used a different laboratory-completeness rule and was treated as a separate sensitivity population. Figure 1 and Table 1 report each analysis population explicitly.

**Figure 1:**
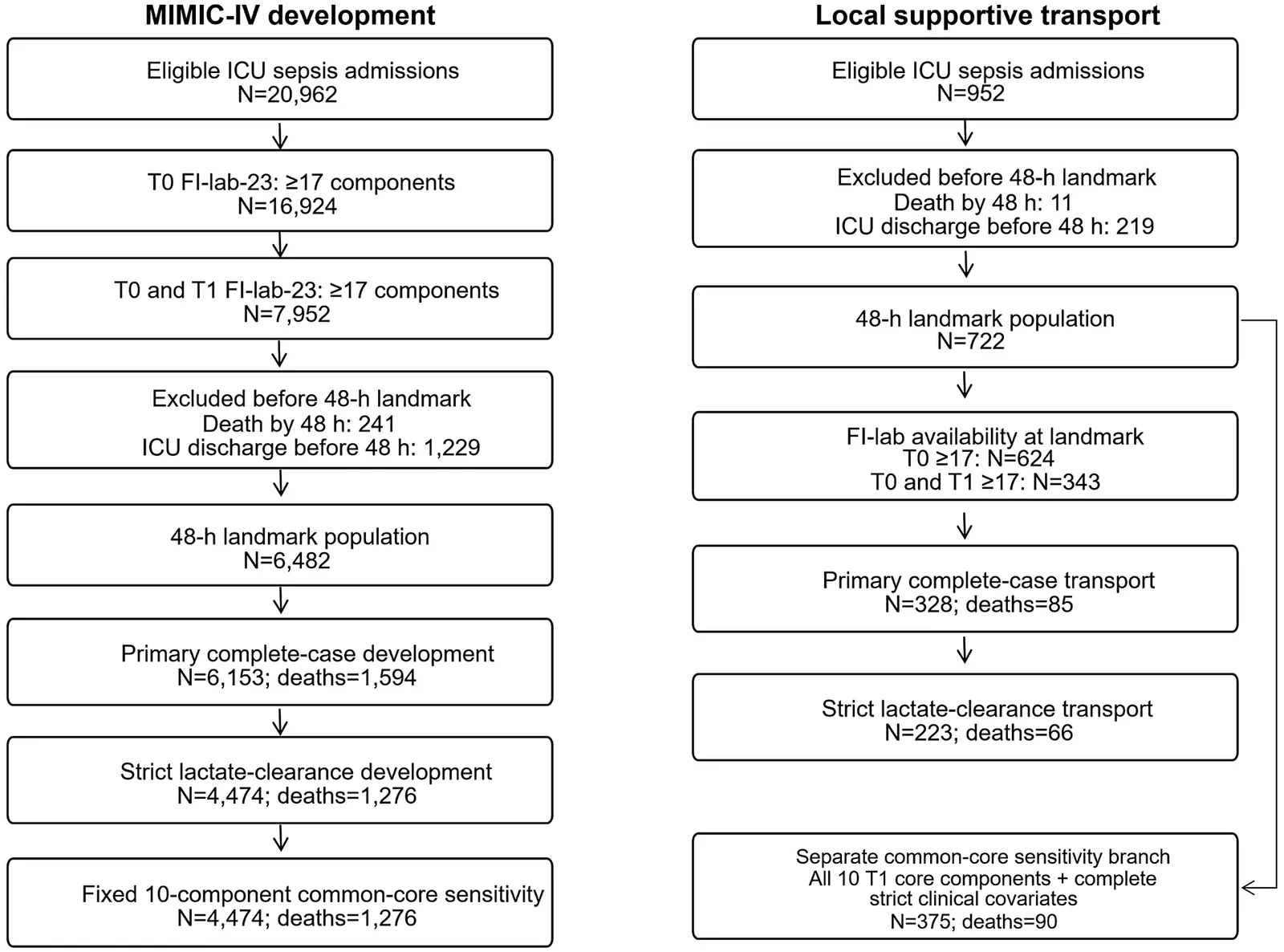
Analysis-specific flow diagram for development and supportive single-center transport cohorts. In the local cohort, the common-core sensitivity analysis forms a separate branch because it uses a different laboratory-completeness criterion while retaining the strict clinical covariates.

**Table 1:** Cohort construction and analytic populations.

Stage	MIMIC-IV development	Local transport
Eligible ICU sepsis admissions	20,962	952
Sufficient T0 FI-lab components	16,924	624 after landmark
Sufficient T0 and T1 FI-lab components	7,952	343 after landmark
Death by 48 h	241	11
ICU discharge before 48 h	1,229	219
Eligible at 48-h landmark	6,482	722
Insufficient T1 FI-lab components after landmark	-	379
Primary extended complete-case cohort	6,153; 1,594 deaths	328; 85 deaths
Missing baseline lactate before primary model	329	15
Strict lactate-clearance cohort	4,474; 1,276 deaths	223; 66 deaths
Additional missing repeat lactate	1,679	105
Separate fixed 10-component common-core sensitivity	4,474; 1,276 deaths	375; 90 deaths

FI-lab availability was assessed at different stages of cohort construction in the two databases: the MIMIC-IV availability counts were derived before landmark exclusions, whereas the local availability counts are reported among the 722 landmark-eligible patients.

### Laboratory windows and FI-lab-23 construction

Baseline measurements (T0) were obtained during 0–24 h after ICU admission, and reassessment measurements (T1) during 24–48 h. The first eligible value recorded within each window was used according to the harmonized extraction algorithm. Absolute neutrophil and lymphocyte counts were used; when only differential percentages were available, absolute counts were calculated as total white blood cell count multiplied by the corresponding percentage divided by 100.

FI-lab-23 comprised 23 routine variables across hematologic, hepatic or nutritional, renal or metabolic, electrolyte, coagulation, and perfusion domains. The same numeric cut-points and units were used in both cohorts after unit harmonization (Table 2). Thresholds were prespecified before outcome modeling and were not optimized against mortality. Routine hematology, chemistry, and coagulation cut-points reflected commonly used adult reference intervals, whereas lactate >2 mmol/L and glucose <70 or >180 mg/dL reflected widely used critical-care clinical conventions. Each available component was coded 1 when outside the prespecified range and 0 otherwise. FI-lab-23 was the number of abnormal components divided by the number measured. At least 17 of 23 components (73.9%) were required. This pragmatic threshold was prespecified to retain broad multiorgan coverage while limiting attrition; because it is less stringent than an approximately 80% completeness convention, sensitivity analyses progressively required 20, 21, 22, and all 23 components. Missing components were excluded from both numerator and denominator. FI-lab-23 was interpreted as an acute laboratory abnormality burden during sepsis and not as chronic or geriatric frailty. MIMIC-IV and the local hospital represent different laboratory environments, so residual inter-assay heterogeneity may remain despite use of common thresholds and harmonized units.

**Table 2:** FI-lab-23 components and prespecified abnormality thresholds.

Component	Unit	Abnormal
White blood cell count	×10^9^/L	<4.0 or >12.0
Absolute neutrophil count	×10^9^/L	<1.5 or >7.5
Absolute lymphocyte count	×10^9^/L	<1.0 or >4.0
Hemoglobin	g/dL	<13.5 in men; <12.0 in women
Platelet count	×10^9^/L	<150 or >450
Alanine aminotransferase	U/L	>40
Aspartate aminotransferase	U/L	>40
Total bilirubin	mg/dL	>1.2
Albumin	g/dL	<3.5 or >5.0
Blood urea nitrogen	mg/dL	<7 or >20
Creatinine	mg/dL	<0.6 or >1.3
Sodium	mmol/L	<135 or >145
Potassium	mmol/L	<3.5 or >5.0
Chloride	mmol/L	<98 or >107
Total calcium	mg/dL	<8.5 or >10.5
Phosphate	mg/dL	<2.5 or >4.5
Magnesium	mg/dL	<1.7 or >2.4
Prothrombin time	s	>14
Activated partial thromboplastin time	s	>40
International normalized ratio	ratio	>1.2
Fibrinogen	mg/dL	<200 or >400
Glucose	mg/dL	<70 or >180
Lactate	mmol/L	>2.0

FI-lab-23 = number of abnormal available components/number of available components. At least 17 components were required. The same prespecified thresholds were applied to both cohorts after unit harmonization. Thresholds were fixed before outcome modeling and were not optimized against mortality; routine hematology, chemistry, and coagulation cut-points reflect commonly used adult reference intervals, whereas lactate >2 mmol/L and glucose <70 or >180 mg/dL reflect critical-care clinical conventions.

### Predictors and model specification

The clinical base model included age, sex, comorbidity burden, admission SOFA score, admission lactate, mechanical ventilation, vasopressor use, and continuous renal replacement therapy during the baseline window. Binary treatment variables were coded 1 for present and 0 for absent. Baseline vasopressor exposure was harmonized as any documented norepinephrine, epinephrine, or dopamine administration during 0–24 h; the same time window and binary drug-class definition were applied in both cohorts, although the underlying EHR medication-capture systems differed. The primary extended model added FI-lab-23 at T1, scaled per 0.05 because this corresponds approximately to one additional abnormal component when about 20 components are measured. The strict secondary model additionally included lactate clearance, calculated as 100 × (lactate at T0 − lactate at T1)/lactate at T0 and scaled per 10% to provide a clinically interpretable increment. Predictors and transformations were fixed before evaluation in the local cohort.

The baseline-only, T1-only, baseline-plus-T1, baseline-plus-ΔFI-lab, and baseline FI-lab × ΔFI-lab interaction models were treated as secondary specification analyses. The T1-focused framework was adopted during revision after the algebraic equivalence of baseline-plus-change and baseline-plus-follow-up models was recognized and the specification results were reviewed; these analyses were used to examine model specification and to reassess the original baseline-anchoring interpretation rather than as multiple independent confirmatory tests. Because baseline-plus-T1 and baseline-plus-ΔFI-lab are algebraically equivalent, identical likelihood-based and predictive performance was expected and was used as a code check. An explicit baseline FI-lab × ΔFI-lab interaction was fitted to test effect modification. The simplified T1-only specification was retained if baseline and interaction terms did not add information.

### Model development and external transport evaluation

Both cohorts were analyzed using logistic regression for the fixed day-28 outcome. This provided one directly transportable prediction equation despite the absence of exact death times in the local data. The MIMIC-IV coefficients and intercept were estimated once and then frozen. For each local patient, predicted risk was calculated from the frozen linear predictor without coefficient refitting or local model updating. The complete equations are reported in Table 3.

**Table 3:** Complete MIMIC-IV prediction model coefficients.

Predictor	Extended *β*	Extended OR (95% CI)	Strict β	Strict OR (95% CI)
Intercept	−5.010943	—	−4.925755	—
Age, per year	0.018442	1.019 (1.014–1.024)	0.022024	1.022 (1.016–1.028)
Male sex	−0.106918	0.899 (0.794–1.016)	−0.099997	0.905 (0.786–1.042)
Comorbidity score	0.074703	1.078 (1.049–1.107)	0.051987	1.053 (1.021–1.087)
Admission SOFA	0.057088	1.059 (1.037–1.081)	0.065483	1.068 (1.043–1.093)
Admission lactate, mmol/L	0.035370	1.036 (1.011–1.062)	0.066836	1.069 (1.039–1.100)
Mechanical ventilation	0.034912	1.036 (0.910–1.178)	−0.028560	0.972 (0.837–1.128)
Vasopressor use	0.081315	1.085 (0.943–1.248)	0.062438	1.064 (0.905–1.252)
CRRT	0.190400	1.210 (0.945–1.548)	0.091709	1.096 (0.845–1.422)
Lactate clearance, per 10%	—	—	−0.034579	0.966 (0.955–0.977)
FI-lab-23 at 24–48 h, per 0.05	0.163650	1.178 (1.149–1.207)	0.142919	1.154 (1.120–1.188)

Extended equation: LP = −5.010943 + 0.018442 × age − 0.106918 × male + 0.074703 × comorbidity + 0.057088 × SOFA + 0.035370 × lactate + 0.034912 × MV + 0.081315 × vasopressor + 0.190400 × CRRT + 0.163650 × (FI-lab_T1/0.05). Predicted risk = 1/[1 + exp(−LP)].

Strict equation: LP = −4.925755 + 0.022024 × age − 0.099997 × male + 0.051987 × comorbidity + 0.065483 × SOFA + 0.066836 × lactate − 0.028560 × MV + 0.062438 × vasopressor + 0.091709 × CRRT − 0.034579 × (lactate clearance/10) + 0.142919 × (FI-lab_T1/0.05). Predicted risk = 1/[1 + exp(−LP)]. Binary predictors are coded 1 when present and 0 when absent.

Development performance included apparent AUC and Brier score. Optimism was estimated with 200 bootstrap samples by refitting each model in the bootstrap sample and evaluating it in both the bootstrap and original data; the mean optimism was subtracted from apparent performance. External performance included AUC, Brier score, calibration-in-the-large estimated as a calibration intercept with the original frozen linear predictor as an offset, calibration slope estimated against that same frozen predictor, observed-to-expected event ratio, and graphical calibration. Grouped calibration used five groups defined by quintiles of frozen predicted risk. Ninety-five percent confidence intervals for external AUC, Brier score, and the observed-to-expected ratio were obtained from 1,000 patient-level bootstrap samples. Decision curve analysis compared the frozen clinical and FI-lab models over risk thresholds of 0.10–0.40 and included patient-level bootstrap uncertainty for the FI-lab net-benefit curve (25–27). It was prespecified as exploratory because the local event count was limited and was not interpreted as evidence of established clinical utility.

### Missing data, measurement intensity, and sensitivity analyses

Primary analyses used complete cases for all predictors required by the relevant model. Because test ordering can be informative, we reported the number of available FI-lab components at T1 and repeated the strict association analysis after adjustment for this count. We additionally restricted analyses to patients with at least 20, 21, 22, or all 23 T1 components. A fixed common-core FI was constructed from ten components with high availability in both cohorts: white blood cell count, hemoglobin, platelet count, blood urea nitrogen, creatinine, sodium, potassium, chloride, total calcium, and glucose. All ten components were required for this analysis. In the local common-core analysis, the same strict clinical adjustment set, including admission lactate and lactate clearance, was retained; the different laboratory-completeness rule produced a separate sensitivity population of 375 patients with 90 deaths rather than a subset of the primary FI-lab-23 cohort. We compared included and excluded landmark patients on age, sex, comorbidity score, SOFA, admission lactate, T1 component count, and mortality. Multiple imputation was not used because most exclusions in the local cohort arose from structurally unmeasured FI-lab components (379 of 394 exclusions), for which missingness was plausibly related to clinician test-ordering behavior rather than missing at random; imputing these unmeasured laboratory values would also change the prespecified observed-component FI construct. We therefore addressed missingness with explicit selection comparisons, component-count adjustment, progressively stricter completeness thresholds, and the fixed common-core analysis. These sensitivity analyses evaluated robustness of the association and do not establish frozen-model performance among patients excluded by the complete-case requirements.

Modified FI analyses that simply removed SOFA-overlapping components were not used to rescue the original change-score claim. Instead, the revised analyses directly addressed the more fundamental concerns by using the 48-h level, fixed thresholds, component-count adjustment, common-core scoring, and frozen-coefficient transport.

### Sample size considerations

The development sample was determined by all eligible MIMIC-IV records and provided 1,594 events in the primary model. The local primary cohort provided 85 events and 243 non-events, and the strict cohort provided 66 events and 157 non-events. Contemporary guidance recommends designing external evaluations for adequate precision of calibration, discrimination, and net benefit rather than relying only on events-per-variable rules (24, 28, 29). The local study was therefore interpreted as a supportive external transport evaluation with potentially imprecise calibration estimates, not as definitive evidence for deployment.

### Statistical analysis

Continuous variables were summarized as medians and interquartile ranges and categorical variables as counts and percentages, except that continuous variables in Table 4 are additionally presented as mean ± standard deviation to match the quantities used for standardized mean difference calculations. Case-mix differences were described with standardized mean differences (SMDs), with absolute values below 0.10 considered negligible, 0.10–0.20 small, 0.20–0.50 moderate, and above 0.50 large. Associations were presented as odds ratios with 95% confidence intervals. Nested development models were compared using likelihood ratio tests and Akaike information criterion. External AUC differences between paired frozen models were estimated using a patient-level paired nonparametric bootstrap so that the same resampled patients contributed predictions from both models. Two-sided *P* values below 0.05 were considered statistically significant for prespecified coefficient tests; secondary specification analyses were interpreted as analytical checks rather than multiple independent confirmatory tests. Performance estimates were interpreted primarily by effect size and uncertainty. Analyses were performed in R 4.5.1 and independently cross-checked in Python 3.11.

**Table 4:** Comparison of included and excluded patients in the local 48-h landmark cohort.

Variable	Included (*N* = 328)	Excluded (*N* = 394)	SMD
Age, years	61.68 ± 16.78	66.86 ± 16.07	−0.315
Male sex	202 (61.6%)	223 (56.6%)	0.102
Comorbidity score	5.07 ± 2.91	4.81 ± 2.82	0.093
SOFA score	7.89 ± 3.88	5.12 ± 3.04	0.797
Admission lactate, mmol/L	2.83 ± 2.57	2.01 ± 1.36^†^	0.398
Available T1 FI-lab components	19.52 ± 1.39	14.42 ± 1.97	2.989
28-day mortality	85 (25.9%)	58 (14.7%)	0.281

† Admission lactate was available in 324 of 394 excluded landmark patients. Continuous variables are presented as mean ± standard deviation to match the calculation of SMDs. The primary cohort excluded 379 patients with fewer than 17 T1 FI-lab components and 15 additional patients with sufficient FI-lab components but missing admission lactate.

### Ethics

MIMIC-IV contains deidentified data and was accessed after completion of the required credentialing and data-use agreement. The local study was approved by the Ethics Committee of The First Affiliated Hospital of Chengdu Medical College (approval number 2026CYFYIRB-BA-045). Informed consent was waived because the study was retrospective and used deidentified data.

## Results

### Cohort construction and complete-case selection

The cohort flow is shown in Figure 1 and Table 1. Of 20,962 eligible MIMIC-IV patients, 16,924 had sufficient T0 FI-lab-23 components and 7,952 had sufficient components at both T0 and T1 for the common specification-analysis population. Among these, 241 died by 48 h and 1,229 were discharged from the ICU before 48 h, leaving 6,482 patients at the landmark. The primary extended model included 6,153 patients with 1,594 deaths (25.9%); the remaining 329 landmark patients lacked admission lactate and had 39 deaths (11.9%). The strict model included 4,474 patients with 1,276 deaths (28.5%); an additional 1,679 primary-cohort patients lacked repeat lactate required for lactate clearance.

The local cohort included 952 patients. Eleven died by 48 h and 219 were discharged from the ICU before 48 h, leaving 722 landmark-eligible patients. Among these, 624 had at least 17 T0 FI-lab components and 343 had at least 17 components at both T0 and T1. Thus, 379 had fewer than 17 T1 components; 15 additional patients with sufficient FI-lab components lacked admission lactate, leaving 328 patients with 85 deaths (25.9%) in the primary transport cohort. The strict cohort included 223 patients with 66 deaths (29.6%) after a further 105 patients without repeat lactate were excluded. The separate 10-component common-core sensitivity population included 375 patients with 90 deaths. Among the 394 landmark patients excluded from the primary transport cohort, 58 died (14.7%). These differences indicated informative complete-case selection and were considered when interpreting transport performance.

### Case-mix comparison

The primary MIMIC-IV and local transport cohorts had similar age, sex distribution, comorbidity score, admission SOFA, admission lactate, mechanical ventilation, vasopressor exposure, CRRT use, and 28-day mortality (absolute SMDs <0.10; Table 5). After harmonizing vasopressor exposure to any documented norepinephrine, epinephrine, or dopamine administration during 0–24 h, use was similar in MIMIC-IV and the local cohort (48.1% vs. 49.4%; absolute SMD 0.027). FI-lab-23 at 24–48 h was moderately lower in the local cohort (0.47 vs. 0.53; absolute SMD 0.223), representing the principal measured case-mix difference relevant to the updated predictor.

**Table 5:** Case-mix comparison between primary development and local transport cohorts.

Variable	MIMIC-IV (*N* = 6,153)	Local (*N* = 328)	Absolute SMD
Age, years	64.00 [52.00–74.00]	63.00 [51.75–74.00]	0.044
Male sex	0.591	0.616	0.052
Comorbidity score	5.00 [3.00–7.00]	5.00 [3.00–7.00]	0.025
SOFA score	8.00 [5.00–11.00]	8.00 [5.00–10.00]	0.006
Admission lactate, mmol/L	2.10 [1.40–3.40]	1.90 [1.30–3.32]	0.015
FI-lab-23 at 24–48 h	0.53 [0.42–0.63]	0.47 [0.37–0.60]	0.223
Mechanical ventilation	3,322 (54.0%)	176 (53.7%)	0.007
Vasopressor use, 0–24 h	2,957 (48.1%)	162 (49.4%)	0.027
CRRT	348 (5.7%)	20 (6.1%)	0.019
28-day mortality	1,594 (25.9%)	85 (25.9%)	0.000

Absolute SMD values are reported. Vasopressor exposure was defined identically in both cohorts as any norepinephrine, epinephrine, or dopamine administration during 0–24 h.

### Specification checks: baseline, change, and 48-h level

The original baseline-anchored interpretation was not supported (Table 6). In the MIMIC-IV strict cohort, the T1-only specification had lower AIC than the baseline-only model (4,818.5 vs. 4,894.6). Adding baseline FI-lab to T1 did not improve fit (AIC 4,820.3), and the conditional baseline association was null (OR: 0.992; *P* = 0.653). Baseline-plus-T1 and baseline-plus-ΔFI-lab produced identical AUC, Brier score, and AIC, as required by algebraic equivalence. The baseline × Δ interaction was not significant (*P* = 0.257).

**Table 6:** Internal and external performance of the frozen 48-h FI-lab models.

Analysis	N/events	AUC	Brier score	Calibration intercept	Calibration slope	O:E
Development extended	6,153/1,594	0.712 apparent; 0.710 corrected	0.171 apparent; 0.171 corrected	—	—	—
Local extended transport	328/85	0.774 (0.717–0.827)	0.160 (0.140–0.182)	0.109 (-0.155 to 0.373)	1.273 (0.902–1.643)	1.074 (0.898–1.246)
Development strict	4,474/1,276	0.720 apparent; 0.717 corrected	0.179 apparent; 0.180 corrected	—	—	—
Local strict transport	223/66	0.782 (0.717–0.844)	0.166 (0.143–0.192)	0.090 (-0.221 to 0.400)	1.293 (0.865–1.721)	1.056 (0.872–1.242)

Development performance was optimism-corrected with 200 bootstrap samples. External AUC, Brier score, and O:E 95% confidence intervals were obtained from 1,000 patient-level bootstrap samples. No multiple-imputation analysis was used.

The local data showed the same pattern. In the extended cohort, T1-only AIC was 325.5, whereas baseline-plus-T1 AIC was 327.5; the baseline term was null after T1 adjustment (OR: 0.989; *P* = 0.880), and the interaction was not significant (*P* = 0.558). In the strict cohort, the corresponding baseline OR was 1.012 (*P* = 0.891), and the interaction *P* value was 0.265. These results led to removal of baseline anchoring from the primary scientific claim and clinical workflow.

### Model development and internal validation

In the primary MIMIC-IV model, each 0.05 higher FI-lab-23 at 24–48 h was associated with 17.8% higher odds of death by day 28 after adjustment for clinical variables (OR: 1.178; 95% CI: 1.149–1.207; *P* < 0.001). A 0.05 increment is approximately one additional abnormal component when about 20 components are available. Adding T1 FI-lab reduced AIC from 6,556.4 to 6,383.3 (likelihood ratio *P* < 0.001) and increased apparent AUC from 0.685 to 0.712. After 200-sample bootstrap correction, AUC was 0.710 and Brier score was 0.171.

In the strict development cohort, which additionally included lactate clearance, the adjusted OR per 0.05 higher T1 FI-lab was 1.154 (95% CI: 1.120–1.188; *P* < 0.001). AIC decreased from 4,911.7 to 4,818.5 and apparent AUC increased from 0.703 to 0.720. Optimism-corrected AUC and Brier score were 0.717 and 0.180, respectively. Full coefficients and intercepts are reported in Table 3 so that individual predictions can be reproduced.

### External transport without refitting

The frozen primary model was applied directly to 328 local patients as the main supportive single-center transport analysis. AUC was 0.774 (95% CI: 0.717–0.827), compared with 0.720 (95% CI: 0.662–0.778) for the frozen clinical model; the paired bootstrap AUC difference was 0.054 (95% CI: 0.017–0.092) (Figure 2, Table 7). Brier score was 0.160 (95% CI: 0.140–0.182). The calibration intercept was 0.109 (95% CI: −0.155 to 0.373), the slope was 1.273 (95% CI: 0.902–1.643), and the observed-to-expected ratio was 1.074 (bootstrap 95% CI: 0.898–1.246). The observed event rate was 25.9% and the mean predicted risk was 24.1%. Grouped calibration used quintiles of frozen predicted risk, with 65–66 patients per group (Figure 3). Because this evaluation included only 85 events and 328 of 722 landmark-eligible patients after informative complete-case filtering, the transport estimates were interpreted as supportive and imprecise.

**Figure 2:**
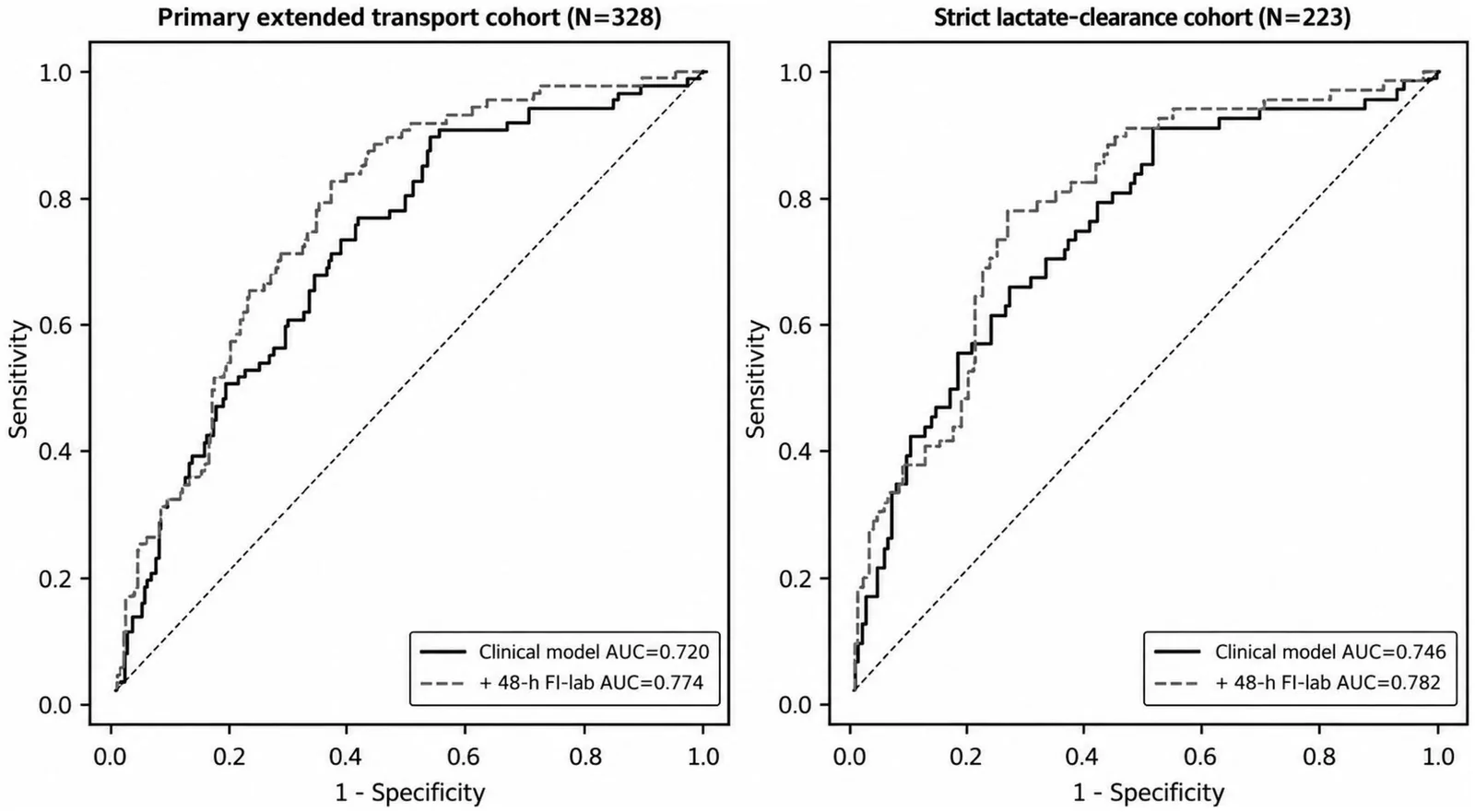
Receiver operating characteristic curves for frozen clinical and 48-h FI-lab models in the local extended and strict cohorts.

**Figure 3:**
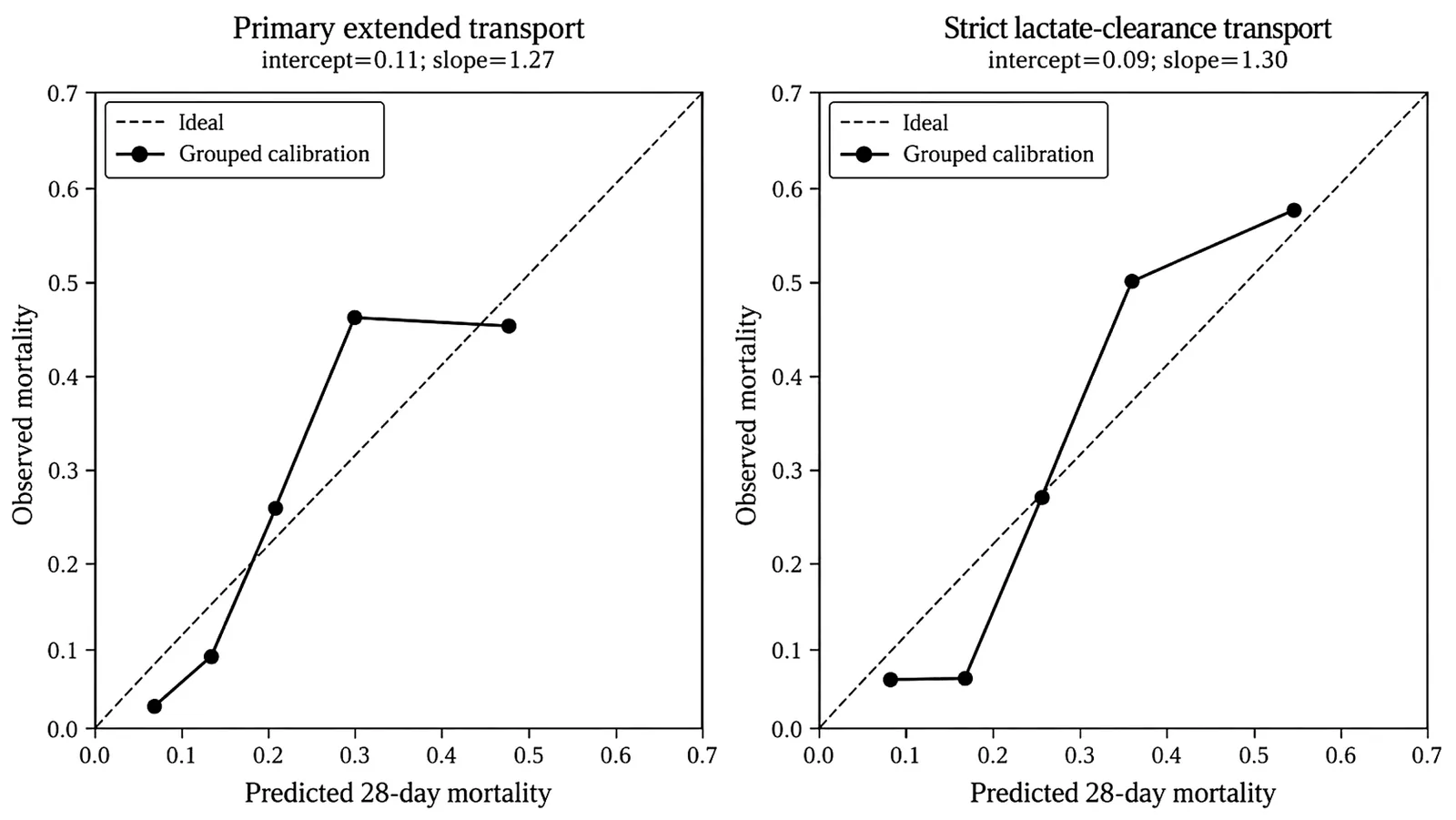
Calibration of frozen 48-h FI-lab models in the local extended and strict cohorts. Grouped calibration used five groups defined by quintiles of frozen predicted risk (65–66 patients per group in the primary cohort and 44–45 per group in the strict cohort); the dotted line denotes ideal calibration.

**Table 7:** Analysis-specific measurement-intensity and common-core sensitivity analyses.

Cohort	Analysis	*N*	Events	Adjusted OR (95% CI)	*P*
MIMIC-IV	Strict-cohort FI-lab-23 level	4,474	1,276	1.154 (1.120–1.188)	<0.001
MIMIC-IV	Adjusted for number of available T1 components	4,474	1,276	1.152 (1.119–1.186)	<0.001
MIMIC-IV	At least 20/23 T1 components available	2,575	808	1.162 (1.117–1.208)	<0.001
MIMIC-IV	At least 21/23 T1 components available	1,233	381	1.213 (1.143–1.287)	<0.001
MIMIC-IV	At least 22/23 T1 components available	410	135	1.272 (1.146–1.412)	<0.001
MIMIC-IV	All 23/23 T1 components available	155	55	1.349 (1.126–1.615)	0.001
MIMIC-IV	Fixed 10-component common-core FI	4,474	1,276	1.092 (1.066–1.119)	<0.001
Local	Fixed 10-component common-core FI	375	90	1.127 (1.029–1.235)	0.010
Local	Strict-cohort FI-lab-23 level	223	66	1.283 (1.119–1.473)	<0.001
Local	Adjusted for T1 testing intensity proxy	223	66	1.303 (1.131–1.502)	<0.001

Analysis populations are not identical. The local common-core analysis retained the strict clinical covariate set, including admission lactate and lactate clearance, but required all 10 common-core laboratory components rather than at least 17 of 23 FI-lab components; therefore, its denominator (*N* = 375) is larger than the primary FI-lab-23 transport cohort (*N* = 328).

In the strict transport cohort, AUC was 0.782 (95% CI: 0.717–0.844), Brier score was 0.166 (95% CI: 0.143–0.192), calibration intercept was 0.090 (95% CI: −0.221 to 0.400), calibration slope was 1.293 (95% CI: 0.865–1.721), and the observed-to-expected ratio was 1.056 (bootstrap 95% CI: 0.872–1.242). The paired AUC difference from the corresponding frozen clinical and lactate-clearance model was 0.036 (95% CI: 0.002–0.069). Grouped calibration used 44–45 patients per predicted-risk quintile. Decision curves were interpreted only over thresholds of 0.10–0.40 and remained exploratory (Figure 4); patient-level bootstrap uncertainty widened at higher thresholds, and the pointwise interval for the FI-lab net-benefit curve included zero at a threshold of 0.40 in both local cohorts. These results do not establish clinical utility and require prospective impact evaluation.

**Figure 4:**
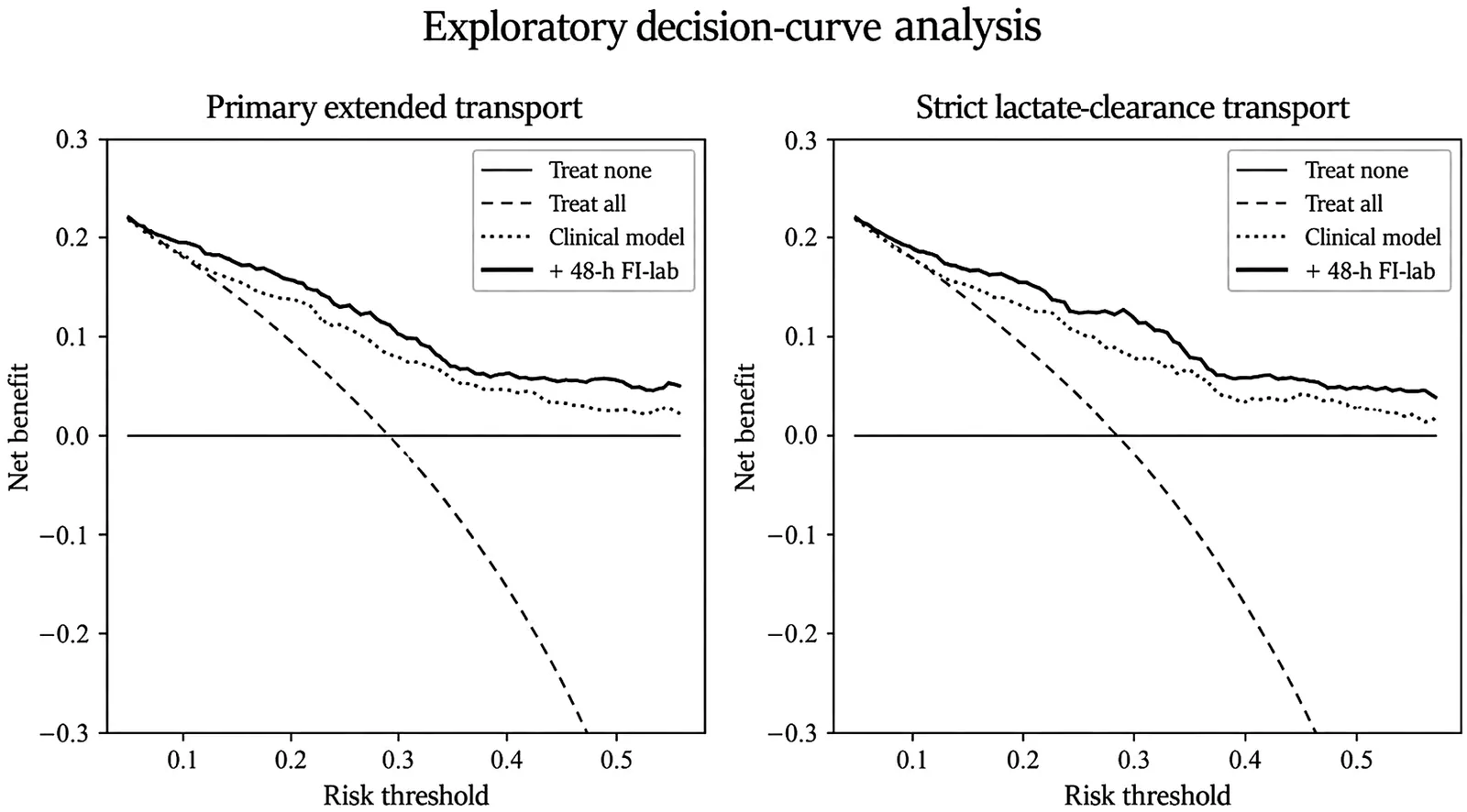
Decision curve analysis of frozen clinical and 48-h FI-lab models in the local extended and strict cohorts. Interpretation was restricted to risk thresholds of 0.10–0.40; bootstrap uncertainty is summarized in the Results.

### Measurement-intensity and common-core sensitivity analyses

The direction of the T1 FI-lab association was preserved after adjustment for the number of measured components in MIMIC-IV (OR: 1.152 per 0.05; 95% CI: 1.119–1.186) and after adjustment for a local testing-intensity proxy (OR: 1.303; 95% CI: 1.131–1.502). Effect magnitude varied as laboratory completeness became more stringent, with MIMIC-IV ORs ranging from 1.162 among patients with at least 20 components to 1.349 among those with all 23. The fixed 10-component common-core FI was associated with mortality in both MIMIC-IV (OR: 1.092; 95% CI: 1.066–1.119) and the local cohort (OR: 1.127; 95% CI: 1.029–1.235) Figure 5, Table 8. In the local landmark population, included patients were younger, had higher SOFA scores, higher measured admission lactate, more available T1 components, and higher mortality than excluded patients (Table 4). These findings confirm selection toward more intensively investigated and more severely ill patients. The sensitivity analyses support directional robustness of the association but do not validate frozen-model performance in the excluded population.

**Figure 5:**
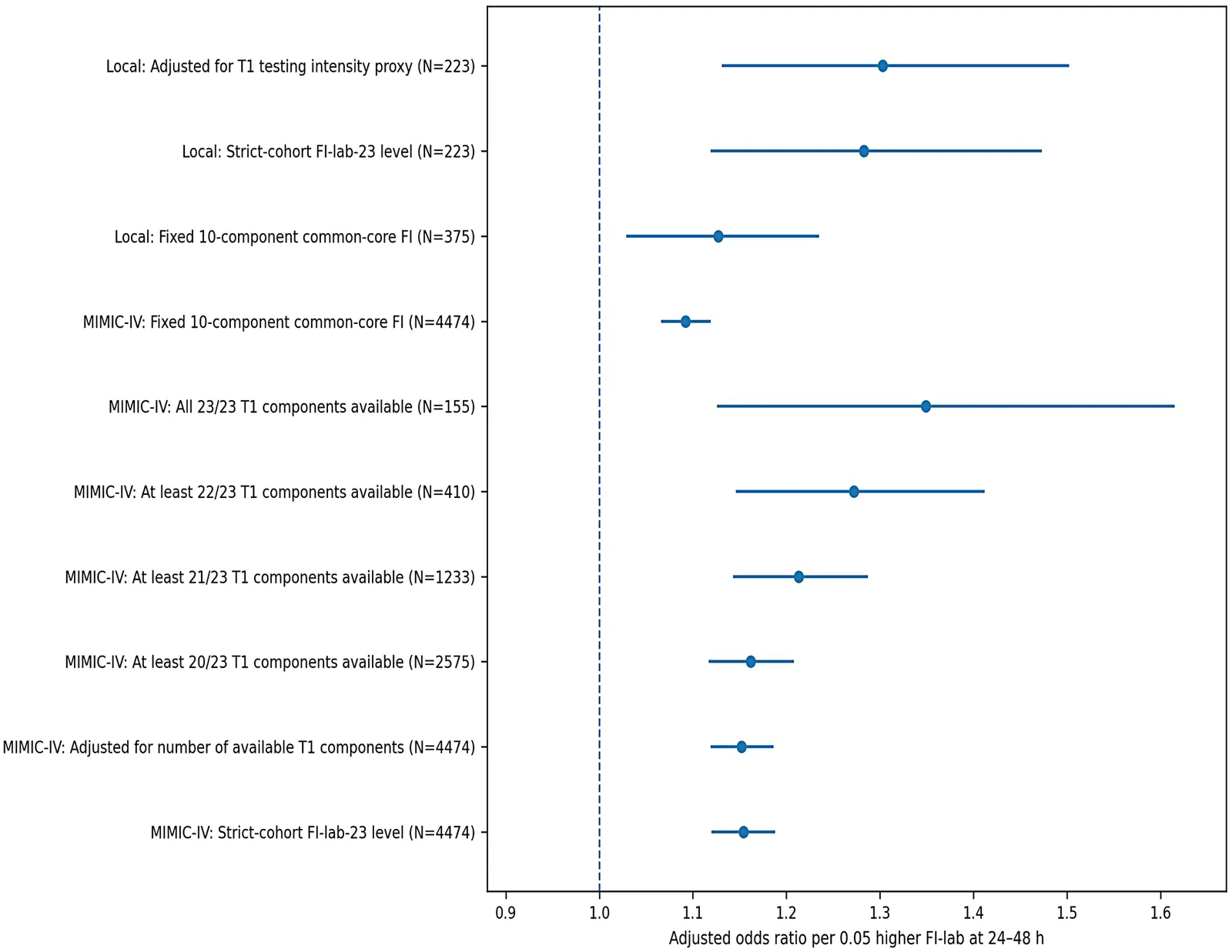
Sensitivity analyses for FI-lab-23 component availability, testing intensity, and the fixed 10-component common-core index.

**Table 8:** Specification checks for baseline, change, and 48-h FI-lab.

Cohort	Specification	AUC	Brier	AIC	Key coefficient
MIMIC-IV strict	Baseline only	0.708	0.182	4,894.6	T0 OR: 1.065; *P* < 0.001
MIMIC-IV strict	48-h level only	0.720	0.179	4,818.5	T1 OR: 1.154; *P* < 0.001
MIMIC-IV strict	Baseline + 48-h level	0.719	0.179	4,820.3	T0 OR: 0.992; T1 OR: 1.158
MIMIC-IV strict	Baseline + delta	0.719	0.179	4,820.3	T0 OR: 1.149; Δ OR: 1.158
MIMIC-IV strict	Baseline + delta + interaction	0.720	0.179	4,821.0	Interaction OR: 1.005; *P* = 0.257
Local extended	Baseline only	0.759	0.163	344.0	T0 OR: 1.171; *P* = 0.009
Local extended	48-h level only	0.790	0.154	325.5	T1 OR: 1.319; *P* < 0.001
Local extended	Baseline + 48-h level	0.790	0.154	327.5	T0 OR: 0.989; T1 OR: 1.326
Local extended	Baseline + delta	0.790	0.154	327.5	T0 OR: 1.312; Δ OR: 1.326
Local extended	Baseline + delta + interaction	0.791	0.153	329.1	Interaction OR: 1.013; *P* = 0.558
Local strict	Baseline only	0.785	0.164	242.8	T0 OR: 1.158; *P* = 0.049
Local strict	48-h level only	0.808	0.157	232.9	T1 OR: 1.283; *P* < 0.001
Local strict	Baseline + 48-h level	0.808	0.157	234.9	T0 OR: 1.012; T1 OR: 1.277
Local strict	Baseline + delta	0.808	0.157	234.9	T0 OR: 1.292; Δ OR: 1.277
Local strict	Baseline + delta + interaction	0.812	0.155	235.5	Interaction OR: 1.031; *P* = 0.265

Baseline + T1 and baseline + Δ are exact reparameterizations and therefore have identical performance. ORs are per 0.05 FI-lab unit. All specifications used the same clinical covariates; strict models also included lactate clearance.

## Discussion

### Principal findings

This study supports two related but distinct findings. First, FI-lab-23 measured during 24–48 h after ICU admission was independently associated with 28-day mortality after adjustment for admission severity and early organ support. Second, adding FI-lab-23 to the prespecified clinical model improved model fit and produced a modest increase in discrimination. When frozen coefficients were transported to the selected local complete-case cohort, discrimination was encouraging, whereas calibration remained imprecisely estimated. The revised analyses did not support the original baseline-anchoring hypothesis: baseline FI-lab added no information once the 48-h level was known, and baseline-by-change interactions were null. The parsimonious interpretation is therefore that the current laboratory abnormality burden at 48 h, rather than a special baseline-anchored change construct, carries the reproducible signal.

### Relation to previous evidence

Deficit-accumulation and laboratory FI studies have shown that a greater burden of abnormal tests is associated with mortality across adult populations (10–13). Sepsis-specific studies subsequently demonstrated associations between baseline FI-lab measures and outcomes in septic shock and older patients (14, 15). Our study extends this literature by shifting the prediction time to 48 h, after initial treatment and early biological evolution, and by publishing a complete transportable equation rather than limiting analysis to refitted associations. Recent small studies of 48-h biomarker reassessment also reported incremental information beyond admission SOFA (16, 17). The present analysis differs by integrating 23 routinely available domains, using a prespecified landmark, internally correcting performance, and applying frozen coefficients to an independent health system.

### Why the baseline-anchored interpretation was withdrawn

The original manuscript interpreted a model containing baseline FI-lab and ΔFI-lab as evidence that baseline vulnerability anchored the effect of deterioration. That interpretation was not justified. Algebraically, baseline plus change is simply a re-expression of baseline plus the follow-up level; identical performance cannot establish a distinct dynamic mechanism. Moreover, the physiological argument that the same increment has different consequences at different baselines is an interaction hypothesis. Explicit interaction tests were not significant in either cohort. Reporting these negative specification checks is important because it separates a clinically appealing narrative from what the data identify.

### Clinical and digital-health interpretation

The revised workflow requires one standardized FI-lab calculation at the 48-h reassessment point. Despite the established FI-lab nomenclature, the score in this acute-care setting summarizes simultaneous laboratory abnormalities across blood counts, hepatic and renal function, electrolytes, coagulation, glucose, and lactate; it should not be interpreted as a measure of chronic geriatric frailty. It is better viewed as an EHR-derived measure of unresolved multisystem laboratory abnormality burden after initial resuscitation. A higher value could prompt structured review of infection control, antimicrobial adequacy, hemodynamics, renal support, fluid balance, coagulation, and metabolic correction. FI-lab is not a treatment trigger by itself and should complement, rather than replace, SOFA, lactate kinetics, and bedside assessment. Mechanical ventilation, vasopressor exposure, and CRRT are prognostic covariates in this model and should not be interpreted as causal treatment effects.

The model is technically simple to implement because all inputs are routinely recorded. The complete equation allows automated calculation and independent validation. However, implementation would require harmonized unit conversion, reliable time-window assignment, transparent handling of missing components, prospective monitoring for calibration drift, and evaluation of whether displaying the prediction changes care or outcomes. Prediction performance alone does not establish clinical utility.

### External transportability and calibration

After harmonization of the 0–24 h vasopressor definition, vasopressor exposure was similar in the development and local transport cohorts, while the local cohort had moderately lower 48-h FI-lab values. The supportive single-center transport evaluation yielded encouraging discrimination, but it was based on only 85 events and on 328 of 722 landmark-eligible patients after informative complete-case filtering. The model modestly underpredicted the average event rate, whereas calibration slopes were above 1 but included the ideal value of 1. The calibration-slope confidence intervals were wide, and grouped calibration was unstable at higher risks. Spectrum restriction and selection may therefore have influenced discrimination. The external analysis is supportive rather than definitive and does not establish broad transportability, implementation readiness, or generalizability (22–24, 31).

### Measurement validity and missingness

A variable-denominator FI can be influenced by which tests are ordered, so part of the observed prognostic signal may reflect clinician-driven testing intensity in addition to physiology. This issue was particularly important in the local cohort: 379 of 394 patients excluded from the primary transport analysis had fewer than 17 T1 components, and the included patients had higher SOFA scores, higher lactate, substantially more measured components, and higher mortality. The revised study mitigated this problem by reporting common thresholds, using the T1 level rather than a difference between changing denominators, adjusting or restricting component availability, and examining a fixed 10-component FI. Consistency in the direction of association across these analyses supports the underlying signal, but informative measurement and non-random test ordering cannot be fully removed retrospectively. These sensitivity analyses do not establish frozen-model performance for patients excluded by the complete-case requirements.

### Strengths

Strengths include the large development cohort, a clinically defined 48-h landmark, explicit correction of the original change-score interpretation, common numeric FI thresholds, internal bootstrap correction, publication of all coefficients and intercepts, frozen-coefficient external transport, quantitative calibration assessment, decision curve analysis, and sensitivity analyses for component availability and a fixed common-core index.

### Limitations

Several limitations remain. First, the retrospective design permits residual confounding and data-quality errors. Second, informative complete-case selection is a central limitation of the transport analysis: only 328 of 722 landmark-eligible local patients entered the primary evaluation, and included patients were younger but more severely ill and more intensively tested than excluded patients. This pattern may affect event prevalence and model performance in less intensively investigated patients, and the sensitivity analyses cannot establish transport performance in the excluded population. Multiple imputation was not used because most exclusions arose from clinician-dependent absence of multiple FI-lab components, making a missing-at-random assumption questionable. Third, the external cohort was single-center and contained only 85 events in the primary analysis and 66 in the strict analysis, producing imprecise calibration estimates; this evaluation should therefore be considered supportive rather than definitive. Fourth, although common thresholds, units, time windows, and vasopressor drug-class definitions were harmonized, inter-laboratory assay differences and residual differences in EHR medication capture may affect transport. Fifth, FI-lab in acute sepsis reflects current laboratory abnormality burden and should not be interpreted as chronic frailty. Sixth, treatment changes before 48 h may be both causes and consequences of the T1 laboratory state, so the model is prognostic rather than causal. Seventh, pathogen, antimicrobial adequacy, and source-control detail were incomplete locally. The local database did not systematically record treatment received at another hospital or ICU before admission to our ICU. In addition, completeness of post-discharge vital-status ascertainment could not be independently verified in the local database. Finally, prospective impact, fairness across subgroups, cost-effectiveness, and calibration monitoring have not been evaluated.

## Conclusions

FI-lab-23 measured at 24–48 h after ICU admission provided updated prognostic information for 28-day mortality in sepsis. The frozen MIMIC-IV model showed encouraging discrimination in a selected single-center complete-case cohort, but calibration and transportability remain uncertain because of limited events and informative laboratory availability. Baseline FI-lab did not add information after the 48-h level, and no baseline-by-change interaction was identified. These findings support further multicenter evaluation of a standardized 48-h FI-lab reassessment model rather than a baseline-anchored dynamic-change construct; the present evaluation is supportive and does not establish implementation readiness.

## Data Availability

The original contributions presented in the study are included in the article/Supplementary Material, further inquiries can be directed to the corresponding author.
